# Stigmatization, Medication Adherence and Resilience Among Recently Diagnosed People Living With HIV/AIDS (PLWHA): A Mixed‐Method Study

**DOI:** 10.1002/puh2.70036

**Published:** 2025-02-24

**Authors:** Emmanuel O. Adesuyi, Cynthia A. Attah, Oluwatosin A. Ige, Confidence C. Attamah, Khadijah A. Jimoh, Opeoluwa O. Olabode, Oluwadamilare Akingbade, Ayobami O. Adeagbo, Rafiat Akinokun, Esther Ilesanmi, Mary I. Oyeleke, Abiodun E. Dairo, Yetunde O. Tola

**Affiliations:** ^1^ Institute of Nursing Research Osogbo Osun Nigeria; ^2^ College of Health and Care Professions Birmingham City University Birmingham UK; ^3^ Medway NHS Foundation Trust Gillingham UK; ^4^ Bedfordshire Hospitals NHS Foundation Trust Luton UK; ^5^ Sussex Partnership NHS Foundation Trust Worthing UK; ^6^ Department of Nursing Science College of Medicine, University of Lagos Nigeria; ^7^ National Institute of Health Research, Lagos Nigeria; ^8^ Department of Nursing, Faculty of Clinical Sciences University of Ibadan Nigeria; ^9^ Faculty of Nursing University of Alberta Edmonton Canada; ^10^ School of Public Health Sciences, Faculty of Health University of Waterloo Ontario Canada

**Keywords:** HIV/AIDS, medication adherence, Nigeria, resilience, stigma, stigmatization

## Abstract

**Aim and Objectives:**

To investigate the level of stigma, medication adherence and resilience among recently diagnosed people living with HIV/AIDS (PLWHA) and explore the relationship between medication adherence, stigmatization and resilience.

**Design/Method:**

This is a convergent‐parallel mixed‐method design involving both qualitative and quantitative research methodologies. The quantitative aspect utilized a cross‐sectional design among 200 PLWHA at the anti‐retroviral therapy clinic of the Lagos University Teaching Hospital, Lagos, Nigeria, whereas the qualitative part entailed semi‐structured, in‐depth interviews of 26 PLWHA. Spearman's rho correlation was used to explore the relationship between medication adherence, stigmatization and resilience, and qualitative data were analysed using thematic analysis.

**Result:**

Four themes emerged from the qualitative analysis, including building resilience, experiences relating to diagnosis, experiences related to treatment and factors facilitating medication adherence. Overall, 113 (57%) experienced a high level of stigma, 149 (76%) reported high medication adherence, and above average 115 (57.2%) demonstrated high resilience.

**Conclusion:**

In this study, PLWHA in Nigeria who recently received their diagnosis experienced a high level of stigma, resilience and medication adherence. However, nearly one‐third of the participants were non‐adherent to medication due to several reasons. This noteworthy proportion of non‐adherence needs to be addressed while improving resilience and reducing stigmatization.

## 1 Introduction

Being diagnosed with HIV is a life‐altering event that presents several challenges, including dealing with a health condition that requires medication and regular medical monitoring, as well as coping with the associated stigma [1]. HIV stigma and discrimination continue to have a significant global impact on the lives of people living with HIV/AIDS (PLWHA). Nearly 8 out of 10 persons receiving HIV care in a US national study experienced at least one form of stigma [2]. The case is worse in Nigeria, where an estimated 3.8 million individuals live with HIV, making it the country with the second‐largest burden of the disease globally [3]. The most recent national data on HIV/AIDS in Nigeria, published by the Joint United Nations Programme on HIV/AIDS, UNAIDS [4], reveal that nearly all individuals living with HIV have faced one or more forms of stigma and discrimination. Stigma continues to be a barrier to physical and mental health among people living with HIV around the world, particularly in vulnerable populations, and has been linked to a lower quality of life as well as difficulties adhering to Highly Active Antiretroviral Therapy (HAART) [5, 6, 7].

Despite significant advancements in HIV medication therapy, including the development of HAART, a major challenge remains non‐adherence to the medication regimen. Recent studies in some parts of Africa, including South Africa and Nigeria, indicate that there are reasonable levels of non‐adherence to medication [8, 9]. It is most challenging for recently diagnosed patients. According to the study conducted by Yu et al. [10], one‐sixth of the participants reported suboptimal medication adherence within the first 6 months.

Resilience has been seen as a positive psychological, behavioural and/or social adaptation in the face of stressors and adversity [11]. Studies have demonstrated the significant effect of resilience on PLWHA [12]. For instance, Fletcher et al. [13] maintain that resilience is vital for PLWHA who have poor adherence to medical regimens. Although evidence on resilience among PLWHA is still emerging, there is limited information available on stigmatization, medication adherence and resilience among people recently diagnosed with HIV/AIDS in Nigeria, which prompted this study.

### 1.1 Aim

This study aims to understand stigmatization, medication adherence and resilience among recently diagnosed PLWHA in Nigeria.

### 1.2 Specific Objectives

1. To investigate the level of stigma, medication adherence and resilience among recently diagnosed PLWHA.
2. To explore the experiences of recently diagnosed PLWHA with stigma, medication adherence and resilience.
3. To investigate how recently diagnosed PLWHA build resilience to HIV stigma.
4. To explore factors facilitating medication adherence among recently diagnosed PLWHA.

## 2 Materials and Methods

### 2.1 Design

Convergent‐parallel mixed‐method design incorporating both qualitative and quantitative research [14] was used in this study to gather varied but complementary data on the connection between medication adherence, building resilience and stigmatization among recently diagnosed PLWHA. A cross‐sectional design was employed for the quantitative component, and semi‐structured in‐depth interviews were used for the qualitative component to investigate the lived experiences of recently diagnosed HIV‐positive individuals.

### 2.2 Participants

All participants in the study comprised any individual 18 years of age or older, who had been diagnosed with HIV within the last 5 years, enrolled in the HIV clinic at Lagos University Teaching Hospital, and can speak English, Yoruba or Pidgin‐English and have given their informed consent. A total of 200 respondents were recruited using a purposive sampling technique, whereas an interview was conducted among 26 PLWHA at the anti‐retroviral therapy clinic of the Lagos University Teaching Hospital, Lagos, Nigeria.

The sample size was calculated using the Taro Yamane formula [15] which is given as *n* = *N*/1 + *N*(e)^2^, where *n* is the sample size, *N* is the population size 335, *E* is the sampling error (0.05), *I* is the constant, *n* = 335/1 + 335 (0.0025), *n* = 182.3, *n* = 182.

Attrition rate = 10% of calculated sample size + the calculated sample size 18.2 + 182 = 200.

### 2.3 Instrument for Data Collection

The quantitative aspect of the study utilized questionnaires for data collection. The questionnaire consists of four sections as described below (see Appendix B): Section A consists of self‐designed questions to assess the socio‐demographic characteristics of the respondents. Section B utilizes the 12‐item HIV stigma scale developed by Reinius et al. [16] to measure the level of stigma experienced by the respondents. The HIV stigma scale assessed stigma across four subscales, including personalized stigma, disclosure concern, concerns about public attitudes and negative self‐image. Cronbach's alpha for the final combination of the 12 items was above 0.7 [16], indicating an acceptable level of reliability. Section C assesses the level of medication adherence of the respondents by using the six‐item Simplified Medication Adherence Questionnaire developed by Knobel et al. [17]. The instrument's Cronbach alpha internal consistency coefficient of the SMAQ was 0.75, indicating an acceptable level of reliability [17]. Section D consists of a 10‐item Connor–Davidson resilience scale developed by Campbell et al. (2009) to measure the respondents’ resilience level. The instrument has been validated and found to be reliable with a Cronbach alpha value of above 0.8 (Wand et al. 2010; [18]). The qualitative aspect of this study utilized semi‐structured interviews. The interview questions were drawn from an extensive literature review and were in line with the research objectives (see Appendix C).

### 2.4 Data Collection

Quantitative data were collected using the questionnaire. Research assistants who are registered nurses with Bachelor of Nursing Science degrees were trained by the research team members on the appropriate administration of the questionnaires and conducting in‐depth interviews. The respondents were approached on their specific clinic days from 4 July 2023 to 30 November 2023. After explaining to them the potential impact of the study, they were offered an information sheet containing details of the study, after which consent was obtained (see Appendix A). The questionnaires were administered in writing, with research assistants available to address any concerns raised by participants. No incentives were offered for participation. Completing the questionnaire took approximately 15 min, whereas interviews lasted 30–45 min. In the qualitative phase of this study, data collection was conducted until 26 interviews were completed, achieving code and meaning saturation. At this point, no new information emerged, and all emerging themes were thoroughly understood, aligning with Hennink et al. [19], who describe data saturation as a key indicator for determining the completion of interviews.

### 2.5 Data Analysis

Descriptive data were analysed, summarized and presented in frequency tables and figures. Qualitative data were analysed through the inductive approach [20]. To ensure rigour in data collection and analysis, open‐ended questions were utilized to ensure credibility. Audit trails were conducted to ensure dependability and transferability. A senior faculty fellow reviewed the themes, participants’ quotes and interpretations to ensure confirmability [21, 22]. The quantitative and qualitative datasets were analysed independently, after which results were compared, interpreted and synthesized during the interpretation of results.

### 2.6 Ethical Consideration

Ethical approval was obtained from the Research Ethics Committee of Lagos University Teaching Hospital with approval number ADM/DSCST/HREC/APP/5830. Participants were assured of their voluntariness, anonymity and confidentiality. The principles of beneficence, non‐maleficence and trust were upheld throughout the study.

## 3 Results

### 3.1 Qualitative Results

#### 3.1.1 In‐Depth Interviews

The interview includes 26 PLWHA, who were receiving care at the Anti‐Retroviral Therapy Clinic of the Lagos University Teaching Hospital, Nigeria.

The mean age of the participants is 39.5 years ± 3.12, of which 18 individuals were diagnosed within 3 years preceding the interview, and 8 were diagnosed more than 3 years prior. Participants are distributed across four geopolitical zones in Nigeria. Detailed characteristics of the participants are available in Table 1. The two major themes that emerged from the analysis include experiences relating to diagnosis and treatment, building resilience and adhering to medication.

**TABLE 1:** Socio‐demographic information of the interview participants.

Variables	Options	Frequency (*n* = 26)
Age	Mean age: 39.5 ± 3.12	
Sex	Female	18
	Male	8
Marital status	Single	10
	Married	12
	Divorced	4
Geopolitical area of origin	Southwest	5
	Southeast	9
	North–Central	5
	South–South	7
Religion	Christianity	16
	Islam	10
Level of education	Primary	4
	Secondary	13
	Tertiary and above	9
Occupation	Trader	9
	Auxiliary nurse	1
	Student	4
	Craftwork	4
	Civil servant	2
	Banking	2
	Driver	4
Who are you living with?	Alone	9
	Family	11
	Children	6
Family monthly household income	Less than #20,000	10
	#20,000–#50,000	6
	#50,000–#100,000	4
	Not working/Earning	6
Year of diagnosis	≤3 years	18
	>3 years	8

##### 3.1.1.1 Theme 1: Experiences Relating to Diagnosis and Treatment

The participants recounted their experiences at the point of being diagnosed with HIV and treatment. Four facets emerged from their experiences elucidated in detail within the subsequent subthemes.

###### 3.1.1.1.1 Disclosure of HIV Status

There was mixed response regarding the disclosure of their HIV status. Many of the respondents recounted how difficult it was for them to disclose their HIV status to even their family members. Some eventually disclosed to selected family or friends, but many did not.

No, I didn't disclose my status to my family because they have soft minds, and I don't want them to start feeling bad and scared because of my health or feeling that I am going to die. It is only one of my friends who has the same status that knows about my condition and her family members… (P10, Female, 26 years, Student)

###### 3.1.1.1.2 Impact of Diagnosis on Their Physical and Mental Health

Participants articulated how they felt around the time of their diagnosis and treatment, unveiling the consequential effects of this experience on their physical and mental well‐being. Some of them stated that they were depressed and felt they were already dead when they broke the news of their diagnosis. Some had eczema, consistent headaches, general body weakness and coughing.

They sent me a message a few years ago, that I should come and do an HIV test when I had Eczema, and they could not find a treatment…they just broke the news. I became very worried and depressed not just about the test result… if it were to be a pregnancy issue, abortion would be done and the problem solved, but this cannot be solved that way. (P15, Female, 57 years, Civil servant)

###### 3.1.1.1.3 Socioeconomic Impact of Diagnosis

Respondents described how being diagnosed with HIV has impacted their socioeconomic status. Many could not continue their jobs, whereas others could no longer get their dream jobs. Some were indecisive about what job they could do given their current condition. Participants highlighted the impact of an HIV diagnosis on their marital relationships, with single individuals expressing apprehension about entering marital commitments, whereas married participants feared potential abandonment by their spouse or significant changes in the dynamics of their relationship.

It has not been easy… I don't know which job I will be able to do. Yes, it affected me, like I have people that want to marry me, but I cannot accept them. Because as a human being, I don't want anything that will cause problems. I don't know how to tell them… (P8, Female, 22 years, Trader)

###### 3.1.1.1.4 Stigma and Its Impact

Participants narrated their ordeals regarding the stigma effect of being diagnosed and treated for HIV. Some expressed the shame they felt when others recognized their status which altered how they interacted with them. Conversely, some participants highlighted how their family members have assisted in concealing their HIV status aiming to mitigate the potential shifts in behaviours resulting from knowledge of their condition.

Even the time I told my bosom friend, and he went around telling people and when I came back, I noticed the way they were doing, how we used to shake hands before, some of them keeping their hands, so when I noticed, I had to call him and he didn't deny, so no problem. (P19, Male, 27 years, Student)

Some articulated the circumstances around them that influence their attitude. These include the fear of stigmatization especially if people see them taking their medications, lack of funds and the exhaustive effect of taking the medications repeatedly.

Ah, initially I took it as normal, I thought the drugs they were giving me would give me another thing, it was not like…you know. I am just tired of taking the drugs. It's not easy, sometimes I forget to take it, so I had to put it where I can see it., you know I'm old. (P11, Female, 87 years, Trader but no longer trading)

###### 3.1.1.1.5 Theme 2: Building Resilience and Adhering to Medications

Participants described several factors that were instrumental in their journey to resilience and medication adherence. Some of the things that stood out from their narratives were the fear of death and hope of survival, family and friends, fear of death, healthcare workers, media, technology and self‐determination.

###### 3.1.1.1.6 Fear of Death and Hope of Survival

Some of the participants recounted how the fear of death and hope of survival were the strongest reasons for their resilience and medication adherence. They were forced to keep taking their medications as prescribed by the doctors to be in good health and stay alive.

Ah! I don't want to die young, that's my motivation, I know if I take my drugs and do things, I will be able to train my children and give them life; that's why I kept coming and taking my drugs. Because I know with that, I can live better… (P17, Male, 39 years, driver)

###### 3.1.1.1.7 Family and Friends

Several respondents emphasized the significance of robust family support as an encouragement for maintaining medication adherence. They delineated the facilitative effect of having understanding and supportive family and friends, noting the ease it brings to their medication adherence routines.

My siblings know I take drugs separately…my dad tells me in their presence, have you used your medicine? don't use that medicine to play o [don't take your medications with levity], that's your life, even my friends used to encourage me, and this gets me going. (P20, Female, 33 years, Civil servant)

###### 3.1.1.1.8 Healthcare Workers

Some of the participants articulated that their continuous pursuit of medical care and adherence to prescribed medication regimens were propelled by the influence and support of healthcare professionals.

So, the staff here at the clinic, were very helpful in the sense that they try to make you not feel down, and they were very supportive, and all that… (P3, Male, 44 years, Craftworker)

###### 3.1.1.1.9 Media, Technology and Self‐Determination

Participants recounted the pivotal role of technology and media platforms in fostering adherence to their treatment plan. They described how their phone alarm and reminder feature effectively facilitated adherence to medication schedules and hospital appointments. Moreover, they articulated how personal resolve and media programmes served as sources of resilience and fortitude.

But actually, I do watch the National Television Authority (NTA) TV station and see the way they take treatment and that it is not a deadly something. It gives me that sense of responsibility and energy to continue. (P18, Female, 52 years, Trader)

## 4 Quantitative Results

Table 2 presents the socio‐demographic characteristics of PLWHA. Participant's mean age is 42.76 ± 11.002, and 57% of them are females, with about 119 (59.1%) being married. About 80.4% of participants had been diagnosed with HIV within the last three years, whereas 19.1% received their diagnosis more than 3 years ago. Additionally, 83.4% commenced anti‐retroviral therapy within the last 3 years.

**TABLE 2:** Socio‐demographic characteristics for quantitative participants.

	Categories	Frequency	Per cent
Sex	Male	86	43.0
	Female	114	57.0
	Total	200	100.0
Marital status	Single	51	25.5
	Married	119	59.5
	Divorced	15	7.5
	Total	185	92.5
Age(years)	20–29	32	16.0
	30–39	50	25.0
	40–49	62	31.0
	50–59	45	22.5
	60–69	9	4.5
	70–79	2	1.0
	Total	200	100.0
Years since HIV diagnosis	≤3 years	160	80.4
	>3 years	39	19.1
	Total	199	99.5
Years since starting anti‐retroviral therapy	≤3 years	166	83.4
	>3 years	33	16.1
	Total	199	99.5

**FIGURE 1:**
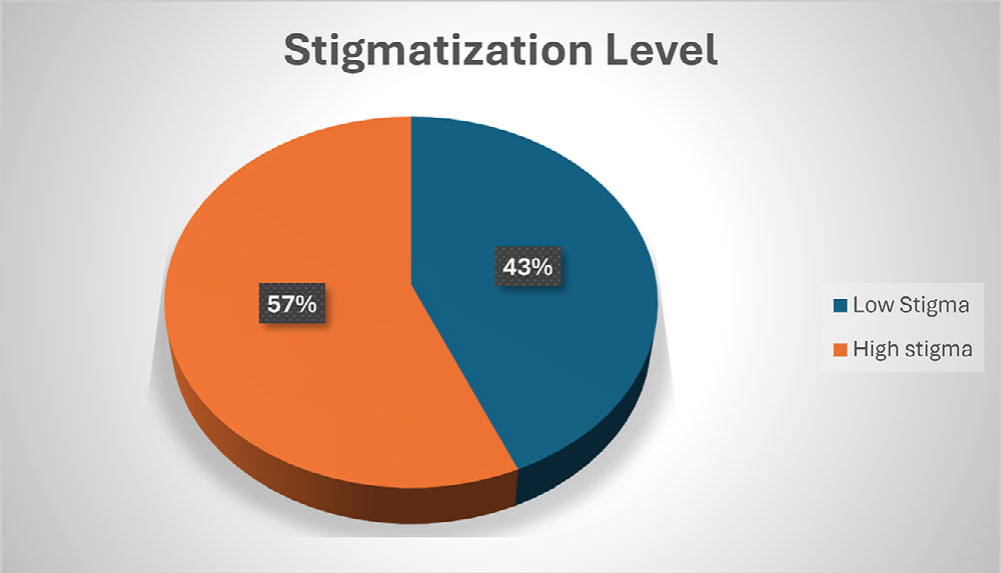
Stigmatization level of PLWHA.

On the Stigma Scale, about 128 (64%) of the participants were careful about disclosing their HIV status due to the risk involved. Half of them found most people uncomfortable around someone with HIV. Few (20.5%) of the respondents believe that people living with HIV have low self‐esteem and feel guilty (see Appendix D, Table 1). The stigma level categorized based on total scores revealed that 113 (57%) experienced high stigma, whereas 87 (44%) experienced low stigma (see Figure 1).

**FIGURE 2:**
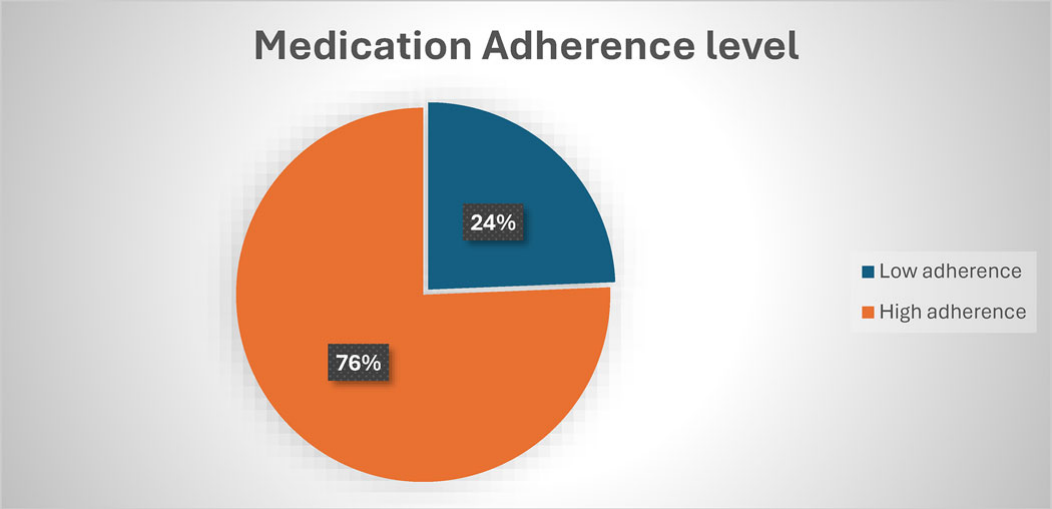
Medication adherence level of PLWHA.

Regarding the participants’ medication adherence, the majority (78%) of the respondents did not stop taking their medicine, including when they felt worse. About 68% never missed their medication during the last week. About 10% reported being careless about taking their medications (see Appendix D, Table 2). The medication adherence categorized based on total scores indicated that 149 (76%) demonstrated high adherence, whereas 48 (24%) reported low adherence (see Figure 2).

**FIGURE 3:**
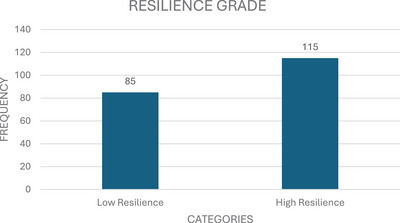
Resilience level of PLWHA.

Data on resilience level showed that more than half of the participants often adapt to changes when they occur and can deal with whatever comes their way. About 66.5% see the humorous side of things with their diagnosis. Overall, 81% believe that they can achieve their goals, even if there are obstacles. More than half tend to bounce back after illness, injury or other hardships. About 62% believe that their status makes them stronger (see Appendix D, Table 3). The resilience levels categorized based on total scores revealed that 85 participants (42.5%) demonstrated low resilience, whereas 115 participants (57.2%) exhibited high resilience (see Figure 3).

### 4.1 Measure of Association

We used Spearman's rho correlation after conducting a preliminary analysis which indicated that the continuous variables violated the assumptions of normality and linearity. There was a very small correlation between adherence to medication and stigmatization of PLWHA (*r* = 0.198 at *p* = 0.829 and 95% CI = −0.155 to 0.191), and a small correlation between adherence to medication and resilience (*r* = 0.111 at *p* = 0.829 and 95% CI = −0.063 to 0.297) and resilience and stigmatization of PLWHA (*r* = .255 at *p* < 0.001 and 95% CI = 0.116–0.383). This is shown in Table 3.

**TABLE 3:** Correlations between adherence to medication, stigmatization of people living with HIV/AIDS (PLWHA) and resilience.

			Resilience	Stigma
Spearman's rho	Medication adherence	Correlation coefficient	0.111	0.019
		Sig. (2‐tailed)	0.198	0.829
		*N*	136	136
	Resilience	Correlation coefficient	1.000	0.255^a^
		Sig. (2‐tailed)		<0.001
		*N*	200	200

^a^ Correlation is significant at the 0.01 level (2‐tailed).

### 4.2 Data Integration

In triangulating the data, we found intersections between quantitative and qualitative aspects of the study. Each method illuminates the findings from the other, enhancing the depth of analysis. Well above half of the respondents in the study's quantitative and qualitative aspects were diagnosed within the last 3 years. Although the survey indicates a one‐third non‐adherence rate among the respondents, the interview highlights factors responsible for non‐adherence, including the fear of stigmatization, medication side effects and financial constraints (with nearly half the respondents living on less than a dollar per day). Although the survey indicates that above half of the participants faced a high level of stigmatization, the interviews provide insight into how this experience impacted their lives. This includes the inability to disclose their status, shame which affects their social interaction, diminished employment prospects, rejection from family, poor medication adherence and low motivation to seek medical care. The survey reports that just above three‐quarters of the participants adhered to their medication, whereas the interview elucidates factors influencing adherence. These include the fear of death, support from family and friends, guidance from healthcare workers, media influence and personal determination. The survey result indicates that approximately two‐thirds of the respondents demonstrated high resilience with the interview offering additional insights into how they build resilience. These include the hope of survival, encouragement from friends and family, religious succour, personal determination, engaging with support programmes, seeking guidance from experienced peers living with HIV/AIDS and accessing supportive media content.

### 4.3 Discussion of Findings

This study explored stigmatization, medication adherence and resilience among PLWHA. The results showed that the respondents were predominantly females, consistent with the findings of Idowu et al. [23] on determinants of family support among PLWHA seeking care in a tertiary hospital in Lagos State, Nigeria. This underscores the elevated HIV prevalence among women in Nigeria and treatment centres concentrated in urban areas [24, 25].

All participants have experienced stigmatization in one way or the other; however, just over half of the respondents reported experiencing a high level of stigmatization, indicating a significant overall level of stigma. This has impacted their lives in various ways, including the inability to disclose their status, shame affecting their social interaction, diminished employment prospects, rejection from family, poor medication adherence and low motivation to seek medical care. Several studies conducted across the two major regions of Nigeria (Southern and Northern regions) support these findings, reporting varying prevalences of stigmatization among PLWHA from 25% to 60% [26, 27, 28, 29]. Similarly, numerous studies across Africa have reported alarmingly high rates of stigma and discrimination of PLWHA underscoring its pervasive and unacceptable prevalence throughout the region [30, 31, 32].

The findings from this study also described respondents’ experiences around the diagnosis of HIV and how it impacted the respondents' physical, mental and socioeconomic status. Many could not continue their jobs, whereas others could no longer get their dream jobs. Some were indecisive about what job they could do, given their current condition. Unmarried individuals voiced concerns about entering marital relationships due to apprehensions tied to their diagnosis, whereas married participants feared significant shifts in their relationship dynamics or potential spousal abandonment. This is consistent with the findings from Kimera et al. [33], which revealed that the PLWHA struggle to redefine future goals and aspirations, mourn missed life opportunities and perceive their future as unattainable due to their HIV status and the associated stigma within their society. The extant literature from Nigeria and sub‐Saharan Africa demonstrates that an HIV diagnosis significantly disrupts several aspects of individuals’ lives, including their healthcare‐seeking behaviour and adherence to management plans [28, 34]. This study highlights the crucial period following diagnosis, characterized by intense emotions, which can significantly affect the overall prognosis of their condition and engagement with care.

Our study discovered that over three‐quarters of the participants adhered to their medication. We found a slight relationship between stigma and medication adherence among PLWHA. It further reveals factors influencing adherence, including the fear of death, support from family and friends, guidance from healthcare workers, media influence and personal determination. Aderemi‐Williams et al. [35] corroborate these findings by reporting high adherence to medication among adolescents living with HIV at a tertiary facility in Nigeria. Conversely, Oluwole et al. [36] in Nigeria and Crowley et al. [37] in South Africa reported low medication adherence among adolescents due to stigma, forgetfulness, medication burden and side effects. This study distinctly broadens the scope by including PLWHA from the ages of 20 and above, offering insights that span working‐age adults and older populations, thereby addressing a wider demographic.

The findings from this research depicted approximately two‐thirds of the PLWHA demonstrated high resilience. The factors that influenced how they built resilience include the hope of survival, encouragement from friends and family, religious succour, personal determination, engaging with support programmes, seeking guidance from experienced peers living with HIV/AIDS and accessing supportive media content. This aligns with the studies of Aransiola et al. [26] and Owolabi et al. [29]. In a follow‐up study by Jimu et al. [30], some of the participants concealed their medication to reduce the likelihood of being rejected by romantic partners. Conversely, some participants in our study openly took their medications as they believed that the pills were saving their lives. The researcher also discovered that some of the respondents had to travel far to get to medical facilities to protect the privacy of their HIV status. This was the extent to which PLWHA at the target population could go to survive the issues relating to their disease condition and associated impact. Research on resilience as a protective factor against adverse health consequences in older gay men living with HIV/AIDS done by Krause [38] and Liboro et al. [12] found that improved mental and neurocognitive health outcomes are linked to higher levels of HIV‐related resilience.

This study highlights stigma as a critical factor influencing the health outcomes of PLWHA, impacting care‐seeking behaviour, medication adherence and resilience‐building. Despite legislative efforts like Nigeria's Anti‐discrimination Act [39], stigma remains pervasive, with evidence from this study showing that many people still fear losing their jobs, relationships and confidence, driven by cultural and systemic barriers [40]. Interventions to reduce stigmatization through awareness programmes should target every stakeholder to ensure that the rights of PLWHA are upheld. Participants of this study drew inspiration from relatable individuals sharing their experiences, facilitating resilience, which enabled them to seek medical assistance and adhere to prescribed medication regimens. Establishing effective peer or support groups could significantly address these concerns by fostering shared understanding, encouragement and emotional support.

### 4.4 Study Limitation

This study was conducted at a tertiary teaching hospital located in Nigeria's former capital city, a region characterized by its cultural and ethnic diversity, with inhabitants from all six geopolitical zones—Southwest, South‐South, Southeast, North‐Central, Northwest and Northeast—drawn by business opportunities or urban lifestyle. Although participants were distributed across these zones, the study's quantitative phase was limited by a relatively small sample size, which may constrain the generalizability of the findings.

## 5 Conclusion and Recommendations

The findings of the study revealed that most respondents consider it risky to disclose their HIV/AIDs status and thus prefer to keep the status confidential due to the fear of stigmatization. This in many ways influenced their medication adherence and how they build resilience. The following recommendations are therefore made: (1) Interventions should be focused on reducing stigmatization at various levels of human endeavours through awareness programmes against stigmatization, and policies to protect the rights of PLWHA in the country. (2) The establishment of peer and social support groups should be encouraged amidst PLWHA by the government and all stakeholders of the health team.

## Author Contributions

Conceptualisation; EOA, CAA, CCA, RA, EI, YOT, OA. Data curation; EOA, KAJ, AOA, OAI. Formal analysis: EOA, KAJ, AOA, YOT. Methodology. EOA, RA, EI, YOT, OA. Project administration; EOA, CAA, KAJ, MIO, AED. Resources: CAA, KAJ. Writing—original draft: EOA, CAA, OAI, CCA, KAJ, OOO, AOA, RA, EI, MIO, AED. Writing—review & editing: EOA, OAI, OOO. Supervision; YOT, OA.

## Conflicts of Interest

The authors declare no conflicts of interest.

## Supporting information



Supporting Information

## Data Availability

Data are available on request.
