# Supplementary material for: Stigmatization, Medication Adherence and Resilience Among Recently Diagnosed People Living With HIV/AIDS (PLWHA): A Mixed‐Method Study
Source: Public Health Chall. 2025 Feb 24;4(1):e70036. doi: 10.1002/puh2.70036 (PMC12039361; doi:10.1002/puh2.70036)
Supplement: Supplementary file 1 — Supporting Information [file PUH2-4-e70036-s001.docx]

**APPENDIX A**

**PARTICIPANT INFORMATION SHEET AND CONSENT FORM**

**Ethical Approval No: ADM/DSCST/HREC/APP/5830**

**Title of Research: Understanding Stigmatization, Medication Adherence, and Resilience among Recently Diagnosed People Living With HIV/AIDS (PLWHA)**

**Name and Affiliation of Research:** This study is being conducted by a research team led by Emmanuel O. Adesuyi, a researcher at the Institute of Nursing Research, Nigeria.

**Introduction:** HIV medication adherence and overcoming the stigma associated with HIV-positive diagnosis and treatment are public health priorities. However, HIV-related stigma has negatively impacted the HIV regime and care consistency among PLWHA, jeopardizing overall health outcomes and HIV epidemic control in Nigeria. This study seeks to understand Stigma, Medication Adherence, and Building Resilience among Recently Diagnosed PLWHA to explore and establish the relationship. This will help to promote tailored care and counselling interventions by Nurses to develop appropriate disease management strategies for this population and reduce the risk of drug resistance and HIV treatment failure.

**Purpose of Research: This study aims** to understand and describe the relationship between building resilience and the level of medication adherence among recently diagnosed people living with HIV/AIDS (PLWHA). The study will investigate the importance of building resilience, providing Nurses with the evidence to assist PLWHA in adopting positive coping mechanisms or adaptation to adversities associated with HIV treatment and medication adherence.

**The procedure of the Research:** The interview session will last for about 40 to 90 minutes while completing the questionnaire will take 10-15 minutes. If you agree to participate in the study, you are requested to complete and sign a copy of the consent form. For the interview or questionnaire, questions will be directed to you which you will be required to answer. There is no right, or wrong answer and you are required to answer the question in all honesty. The interviews will be recorded, and anonymity and confidentiality will be maintained.

**Potential Benefits:** No compensation will be given for participating in this study. The study's findings will help promote tailored care and counselling interventions by Nurses to develop appropriate disease management strategies for this population, reducing the risk for drug resistance and HIV treatment failure.

**Potential Risks:** This study will not cause any discomfort, pain, or harm to you.

**Confidentiality**: All information collected from this study will not be traceable to you as your name will not be required and any other personal identifier.

**Willingness to participate:** Your participation in this research is entirely voluntary and you may refuse to participate or withdraw at any time. No compensation will be given for this study.

**What Happens to Research Participants and Communities When the Research is Over** The results of this study will be communicated to you and the public through social media, conferences, and peer-reviewed publications. There is no conflict of interest.

**Statement of Person Obtaining Informed Consent:**

I have fully explained this research to the respondent and given sufficient information, including potential risks and benefits to enable the respondent to make an informed decision.

**___________________ _____________________**

Date Signature

**Statement of Person Giving Informed Consent:**

I have read the description of the research. I understand that my participation is voluntary. I know enough about the purpose, methods, risks, and benefits of the research study to judge that I want to take part in it. I understand that I may freely stop being part of this study at any time. I have received a copy of this consent form to keep for myself.

**___________________ _____________________**

Date Signature

**For further enquiry,**

**Researcher’s contact:**

Emmanuel O. Adesuyi

Mobile contact: +447539688355

Email: [emmanueladesuyi12@gmail.com](mailto:emmanueladesuyi12@gmail.com), [eoadesuyi@inrnigeria.org](mailto:eoadesuyi@inrnigeria.org)

Institute of Nursing Research Nigeria.

**APPENDIX B**

**QUESTIONNAIRE FOR SURVEY**

**SECTION A: SOCIO-DEMOGRAPHIC DATA**

1. Age as at last birthday________________

2. Sex: Male (  ) Female ( )

3. What month and year were you diagnosed of HIV?

4. When did you start your Anti-retroviral drug?

5. Marital status a) Single b) Married c) Divorced

**SECTION B: LEVEL OF STIGMA**

***Answer yes or no to the questions.***

| Sub scale | Question | Response | |
| --- | --- | --- | --- |
|  |  | Yes | No |
| Personalised stigma | People I care about stopped calling after learning I have HIV |  |  |
|  | I have lost friends by telling them I have HIV |  |  |
|  | Some people avoid touching me if they know I have HIV |  |  |
| Disclosure concerns | Telling someone I have HIV is risky |  |  |
|  | I work hard to keep my HIV a secret |  |  |
|  | I am very careful who I tell that I have HIV |  |  |
| Concerns about public attitudes | Most people believe a person who has HIV is dirty |  |  |
|  | Most people are uncomfortable around someone with HIV |  |  |
|  | People with HIV are treated like outcasts |  |  |
| Negative self-image | I feel guilty because I have HIV |  |  |
|  | I feel I’m not as good a person as others because I have HIV |  |  |
|  | People’s attitudes about HIV make me feel worse about myself |  |  |

**SECTION C: MEDICATION ADHERENCE**

***Tick the most appropriate answer***

1. Do you ever forget to take your medicine?  a) Yes   b) No

2. Are you careless at times about taking your medicine? a) Yes   b) No

3. Sometimes if you feel worse, do you stop taking your medicines? a) Yes   b) No

4. Thinking about the last week, how often have you missed your medication? a) Never b) 1-2 times c) 3-5 times d) 6-10 times e) >10 times

5. Did you forget to take your medication at the weekend? a) Yes   b) No

6. In the last three months, how many times have you missed your medication?  a) less than 2 days b) greater than 2 days

**SECTION D: LEVEL OF RESILIENCE**

***Choose the answer that best describe your opinion***

| S/N | Questions | Not true | Rarely true | Sometimes true | Often true | Mostly true |
| --- | --- | --- | --- | --- | --- | --- |
| 1 | I am able to adapt when changes occur. |  |  |  |  |  |
| 2 | I can deal with whatever comes my way. |  |  |  |  |  |
| 3 | I try to see the humorous side of things with my diagnosis |  |  |  |  |  |
| 4 | Having to cope with HIV can make me stronger. |  |  |  |  |  |
| 5 | I tend to bounce back after illness, injury or other hardships |  |  |  |  |  |
| 6 | I believe I can achieve my goals, even if there are obstacles. |  |  |  |  |  |
| 7 | Under pressure, I stay focused and think clearly. |  |  |  |  |  |
| 8 | I am not easily discouraged by failure. |  |  |  |  |  |
| 9 | I think of myself as a strong person when dealing with life’s challenges and difficulties. |  |  |  |  |  |
| 10 | I am able to handle unpleasant or painful feelings like sadness, fear, and anger. |  |  |  |  |  |

**APPENDIX C**

**Interview Guide for Qualitative Study**

**Demographic data**

Kindly provide the information below. Confidentiality and anonymity are assured. The information will be used for the study alone.

1. Age: ___________ years

2. What date and year were you diagnosed with HIV?

3. 2. Marital Status Single (  )   Married (  )   Widowed (  )  Divorced (  ) Separated (   )

4. 3. Ethnic group Yoruba (  )   Igbo (  )   Hausa (  )   Others please specify ____________

5. 4. Religion Christianity (  )   Islam (  ) Others please specify ____________

6. Level of education

7. No formal education ( ) Primary (   )   Secondary (   )   Tertiary or above (   )

7. Occupation ________________

8. Who are you living with? ________________

9. Family monthly household income Less than #20,000 (  )   #20,000- #50,000 (  ) #50,000-#100,000 (  )  100,000-150,000  (  )  Above #150,000 (

**Specific Interview Questions**

1. How did you feel when you were diagnosed with HIV?

2. How did you go about telling your family member about your diagnosis?

3. How did your loved ones react to accepting you with your current diagnosis?

4. Do you think your family members have been supportive? And why?

5. Do you think you can live a fulfilled life with your diagnosis? And why?

6. What do you think about taking drugs every day?

7. How did you build strength and courage to seek medical attention?

**APPENDIX D**

**Table 1. STIGMA**

| Variable Categories | | Frequency | Percent |
| --- | --- | --- | --- |
| Deprived of love from loved one due to HIV status | Yes | 16 | 8.0 |
|  | No | 184 | 92.0 |
|  | Total | 200 | 100.0 |
| Loss friends by telling them of my HIV status | Yes | 15 | 7.5 |
|  | No | 185 | 92.5 |
| Touching avoidance by the people | Yes | 14 | 7.0 |
|  | No | 185 | 92.5 |
|  | Total | 199 | 99.5 |
| Telling someone I have HIV is risky | Yes | 131 | 65.5 |
|  | No | 68 | 34.0 |
|  | Total | 199 | 99.5 |
| Keeping my status confidential | Yes | 134 | 67.0 |
|  | No | 65 | 32.5 |
|  | Total | 199 | 99.5 |
| Careful of who I tell about my HIV status | Yes | 128 | 64.0 |
|  | No | 70 | 35.0 |
|  | Total | 198 | 99.0 |
| Most people believe a person who has HIV is dirty | Yes | 60 | 30.0 |
|  | No | 137 | 68.5 |
|  | Total | 197 | 98.5 |
| Most people are uncomfortable around someone with HIV | Yes | 100 | 50.0 |
|  | No | 97 | 48.5 |
|  | Total | 197 | 98.5 |
| People with HIV are treated like outcasts | Yes | 76 | 38.0 |
|  | No | 121 | 60.5 |
| Feel Guilty | Yes | 49 | 24.5 |
|  | No | 148 | 74.0 |
|  | Total | 197 | 98.5 |
| Low self esteem | Yes | 41 | 20.5 |
|  | No | 156 | 78.0 |
|  | Total | 197 | 98.5 |
| People’s attitudes about HIV make me feel worse about myself | Yes | 83 | 41.5 |
|  | No | 114 | 57.0 |
|  | Total | 197 | 98.5 |

**Table 2. MEDICATION ADHERENCE**

| Variable Categories | | Frequency | Percent |
| --- | --- | --- | --- |
| Do you ever forget to take your medicine | Yes | 41 | 20.5 |
|  | No | 156 | 78.0 |
|  | Total | 197 | 98.5 |
| Are you careless at times about taking your medicine | Yes | 20 | 10.0 |
|  | No | 176 | 88.0 |
|  | Total | 196 | 98.0 |
| Sometimes if you feel worse, do you stop taking your medicines | Yes | 3 | 1.5 |
|  | No | 193 | 96.5 |
|  | Total | 196 | 98.0 |
| Thinking about the last week, how often have you missed your medication | Never | 136 | 68.0 |
|  | 1-2 times | 49 | 24.5 |
|  | 3-5 times | 11 | 5.5 |
|  | Total | 196 | 98.0 |
| Did you forget to take your medication in the weekend | Yes | 31 | 15.5 |
|  | No | 164 | 82.0 |
|  | Total | 195 | 97.5 |
| In the last three months, how many times have you missed your medication | ≤ 2 days | 32 | 16.0 |
|  | ≥ 2 days | 24 | 12.0 |
|  | Total | 56 | 28.0 |

**Table 3 LEVEL OF RESILIENCE**

| Variable | Not sure  Freq (%) | Rarely true  Freq (%) | Sometimes true  Freq (%) | Often true  Freq (%) | Mostly true  Freq (%) |
| --- | --- | --- | --- | --- | --- |
| I am able to adapt when changes occur | 7(3.5) | 5(2.5) | 10(5) | 69(34.5) | 109(54.5) |
| I can deal with whatever comes my way | 1(0) | 3(1.5) | 12(6) | 54(27) | 130(65) |
| I try to see the humorous side of things with my diagnosis | 4(2) | 1(0.5) | 21(10.5) | 41(20.5) | 133(66.5) |
| Having to cope with HIV can make me stronger | 1(0.5) | 0.0 | 14(7) | 61(30.5) | 124(62) |
| I tend to bounce back after illness, injury or other hardships | 3(1.5) | 5(2.5) | 18(9) | 72(36) | 102(51) |
| I believe I can achieve my goals, even if there are obstacle | 8(4) | 3.0 | 8(4) | 30(15) | 162(81) |
| Under pressure, I stay focused and think clearly | 1(0.5) | 5(2.5) | 20(10) | 88(44) | 86(43) |
| I am not easily discouraged by failure | 3(1.5) | 2(1) | 22(11) | 52(26) | 121(60.5) |
| I think of myself as a strong person when dealing with life’s challenges and difficulties | 1(0.5) | 1(0.5) | 8(4) | 64(32) | 126(63) |
| I am able to handle unpleasant or painful feelings like sadness, fear, and anger | 5(2.5) | 1(0.5) | 21(10.5) | 79(39.5) | 94(47) |
